# Transcriptional regulation of miR528-PPO module by miR156 targeted SPLs orchestrates chilling response in banana

**DOI:** 10.1186/s43897-024-00115-1

**Published:** 2025-01-10

**Authors:** Xiangjin Kong, Kuan Peng, Youxia Shan, Ze Yun, Tamas Dalmay, Xuewu Duan, Yueming Jiang, Hongxia Qu, Hong Zhu

**Affiliations:** 1 Guangdong Provincial Key Laboratory of Applied Botany, Key Laboratory of South China Agricultural Plant Molecular Analysis and Genetic Improvement, South China Botanical Garden, Chinese Academy of Sciences, 510650 Guangzhou, China; 2 South China National Botanical Garden, Guangzhou, 510650 China; 3 University of Chinese Academy of Sciences, Beijing, 100049 China; 4 School of Biological Sciences, University of East Anglia, Norwich, UK

**Keywords:** miR156, MaSPL4, miR528, Banana, Chilling response

## Abstract

Banana is sensitive to cold stress and often suffers from chilling injury with browning peel and failure to normal ripening. We have previously reported that banana chilling injury is accompanied by a reduction of miR528 accumulation, alleviating the degradation of its target gene *MaPPO* and raising ROS levels that cause peel browning. Here, we further revealed that the miR528-MaPPO cold-responsive module was regulated by miR156-targeted SPL transcription factors, and the miR156c-MaSPL4 module was also responsive to cold stress in banana. Transient overexpression of miR156c resulted in a more severe chilling phenotype by decreasing the expression of *MaSPL4* and miR528. Conversely, the browning was alleviated in STTM-miR156c silencing and OE-*MaSPL4* samples. Furthermore, DNA affinity purification sequencing and *MaSPL4*-overexpressing transcriptome jointly revealed that MaSPL4 may mediate the transcription of genes related to lipid metabolism and antioxidation, in addition to the miR528-MaPPO module, demonstrating MaSPL4 as a master regulator in the fruit cold response network. In summary, our results suggest that the miR156c-MaSPL4 module can mediate the chilling response in banana by regulating the miR528-MaPPO module and multiple other pathways, which provides evidence for the crosstalk between TFs and miRNAs that can be used for the molecular breeding of fruit cold tolerance.

**Supplementary Information:**

The online version contains supplementary material available at 10.1186/s43897-024-00115-1.

## Core

This study demonstrates that in banana, the miR156-targeted SPLs regulate fruit chilling injury through transcriptional regulation of miR528-PPO module, and MaSPL4 is a key regulator of fruit cold response network. This finding provides a reference for molecular breeding to improve the cold tolerance of fruits and enhance the postharvest quality of horticultural products.

## Gene & accession numbers

Sequence information of banana genes in this study can be found in the banana-genome-hub database (https://banana-genome-hub.southgreen.fr/). The accession numbers can be found in Supplemental Data Set S3.

## Introduction

Cold stress, including chilling (0℃ to 15℃) and freezing (below 0℃), adversely affects plant growth, development, yield and postharvest quality (Ding et al. 2019; Valenzuela et al. 2017). Cold stress mainly affects the growth and development of plants by damaging cell membranes and proteins and degrading cell walls, which even leads to the death of plants (Khademi et al. 2014; Lyons 1973; Zhang et al. 2010). The molecular mechanisms of cold stress have been well-studied in model plants such as *Arabidopsis*, rice and maize (Ding et al. 2019). However, the mechanism of cold stress in non-model plants, especially in horticultural crops, remains unclear.

Bananas, an important horticultural crop, are the most produced and traded fruit in the world, which are also consumed as a staple food in some countries. As a tropical and subtropical fruit, bananas are very sensitive to low temperatures. Bananas exposed to temperature below 13℃ may suffer from browning, pitting, loss of flavor, hardening of the central placenta and failure to ripen (Khademi et al. 2019; Promyou et al. 2008; Zhu et al. 2018). Several studies have revealed the mechanism of abnormal banana softening and reduced flavonoid content caused by cold stress (Song et al. 2022a; Song et al. 2022b), but the mechanism of peel browning is still unclear.

MicroRNAs (miRNAs) are a class of endogenous small noncoding RNAs that negatively regulate the expression of their target genes by guiding mRNA degradation or translational repression via sequence complementarity. miRNA plays an important role in plant growth and development, abiotic and biotic stress (Song et al. 2019). In the past decade, several miRNAs have been found to be involved in cold stress. For example, miR394 is significantly induced by cold stress which targets *leaf curling responsiveness* (*LCR*) gene that encodes an F-box protein. miR394 overexpressing and *lcr* mutants have stronger cold tolerance, while *LCR* overexpressing plants show a more cold-sensitive phenotype than the wild type (Song et al. 2016). Similarly, miR319 expression is decreased under low-temperature stress, while its target genes *OsPCF5*, *OsPCF6*, *OsPCF8* and *OsTCP21* are significantly induced. Overexpressing miR319 induces cold tolerance, and decreased miR319 target expression also leads to increased cold tolerance in rice (Wang et al. 2014a; Yang et al. 2013). miR408 is another miRNA that has been shown to respond to cold stress. miR408 overexpressing plants show greater cold tolerance by enhancing cellular antioxidant levels, while miR408 knockout plants show more cold sensitivity compared to the wild-type (Ma et al. 2015). In addition, miR402 has been shown to positively regulate seed germination in *Arabidopsis* under cold stress by targeting *DML3*, which is involved in DNA methylation. Both miR402 overexpressing and *dml3* mutant plants have earlier seed germination at low temperature than the wild type plants (Kim et al. 2010).

The SQUAMOSA-PROMOTER BINDING PROTEIN-LIKE (SPL) transcription factor was first discovered in *Antirrhinum majus*, which contains a highly conserved DNA binding domain (SQUAMOSA promoter binding protein, SBP domain) consisting of 70 to 80 amino acids (Klein et al. 1996). The SBP domain is involved in the transport of SPL proteins into the nucleus, and specifically binds to the GTAC core motifs of target gene promoter to regulate gene expression (Birkenbihl et al. 2005; Kropat et al. 2005; Liang et al. 2008). Furthermore, most SPL proteins are also regulated by miR156, the most conserved miRNA in plants, forming a miR156-SPL module to regulate the expression of downstream genes (Wang and Wang 2015). The miR156-SPL module controls a variety of agronomic traits, such as plant size, pigment synthesis, tuber enlargement, fruit softening and secondary metabolism synthesis (He et al. 2022). However, there are few studies on the regulation of cold stress by miR156-SPL module.

In our previous study, we found that miR528-PPO module is an important regulatory factor leading to banana browning under cold stress (Zhu et al. 2020). However, the upstream regulatory network of the miR528-PPO module in bananas has not been revealed. Here, we identified 58 *SPL* genes in the banana genome, of which 40 *SPL*s could be targeted by miR156 and most were down-regulated under low temperature stress. Then, we demonstrated that multiple miR156-targeted *SPL*s can bind to the promoter of *MIR528* and activate miR528 expression. Transient silencing of miR156c and overexpression of *MaSPL4* in banana peel could delay the degree of chilling injury, while overexpression of miR156c had the opposite effect. Further, DNA affinity purification sequencing (DAP-seq) generated a genome-wide binding site map for MaSPL4, uncovering the miR156/SPL regulatory network of downstream genes that orchestrates banana chilling response.

## Results

### miR528 enhances cold tolerance in banana

**Fig. 1:**
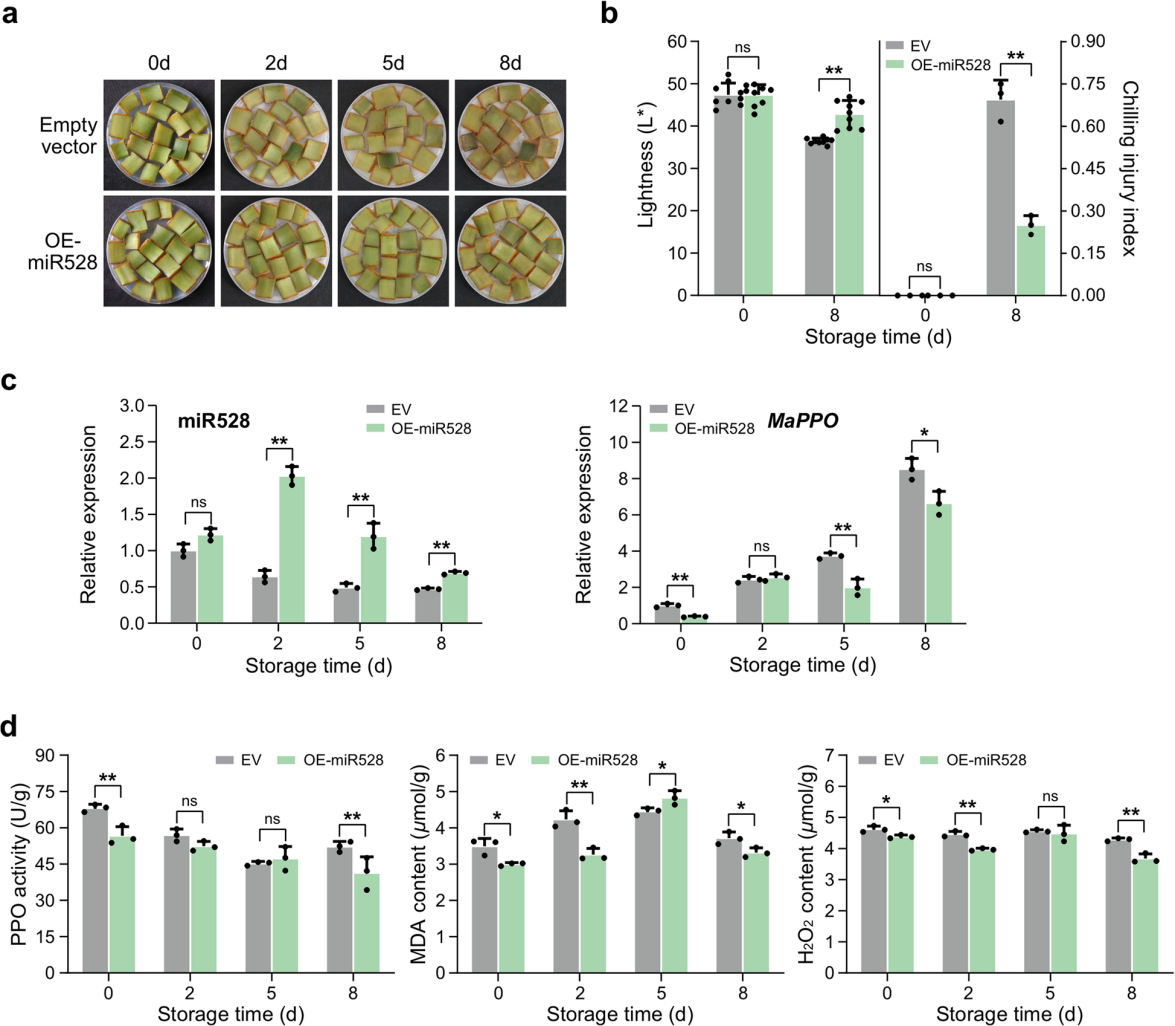
miR528 enhances cold tolerance in banana. **a-b** Phenotype, lightness (L*) and chilling injury index of transient OE-miR528 and control banana peel slices under cold stress. **c** miR528 and *MaPPO* expression in the control and transient transgenic samples. U6 and *Ribosomal protein S2* (*RPS2*) were used as internal references for miRNA and coding genes, respectively. **d** Changes in the PPO activity, MDA content and H_2_O_2_ level in control and transient transgenic samples. Error bars indicate the standard deviation (SD) of three or nine biological replicates (*n* = 3 or *n* = 9) and asterisks indicate significant differences between control and transient transgenic samples at each time point, as determined by two-tailed unpaired Student’s *t*-test (*, *P* < 0.05; **, *P* < 0.01, ns, not significant)

Based on our previous report that miR528-PPO module plays an important role in banana peel browning under cold stress, we further verified the function of miR528 using banana peel slices overexpressing miR528 (Fig. 1). Two days after *Agrobacterium* infection, the peel slices were placed at 6℃. Over a period of eight days, the control samples gradually turned brown, especially at the later stage. By contrast, the OE-miR528 samples showed strong cold tolerance without obvious browning until 8 d (Fig. 1a). On the eighth day under cold stress, the lightness (L*) of the OE-miR528 slices was significantly higher than that of the control group, while the chilling injury index was significantly lower (Fig. 1b). The accumulation of miR528 in the OE-miR528 slices was about 1.5 ~ 4 times higher than that in the control. Correspondingly, the expression level of miR528-targeted *PPO* gene in the OE-miR528 was 25 ~ 50% lower than that in the control (Fig. 1c). In addition, PPO activity, the content of MDA and H_2_O_2_ level that indicates the level of membrane lipid peroxidation also well explained the phenotypes of the corresponding samples (Fig. 1d). All these results demonstrated that miR528 could reduce ROS metabolism and membrane damage in the peel by inhibiting *PPO* expression and enzymatic activity, thereby enhancing cold resistance of banana.

Given these results and the previously reported important and extensive functions of miR528 in monocotyledonous plants, we further set out to explore how miR528 itself is regulated to extend this regulatory pathway. We first mapped the miR528 precursor (pre-miR528) sequence to the banana genome to locate the 1.5 kb upstream region as the *MIR528* promoter. Next, the *MIR528* promoter was cloned and sequenced for cis-element analysis (Supplemental Fig. S2, Supplemental Data Set S2). Notably, the *MIR528* promoter region contained up to eight copper-responsive GTAC motifs that are also the binding sites of transcription factors belonging to the SPL family, which prompted us to explore which members of the SPL family could directly regulate miR528 expression.

### Analysis of banana *SPL* family members

To comprehensively identify the *SPL* gene family members in banana, both BLAST analyses and HMM searches were performed to search against the banana genome database (v4.3). A total of 58 *MaSPL* genes were obtained after manual check to eliminate redundant and low-similarity sequences, and further conserved domain validation. They were renamed from *MaSPL1* to *MaSPL58* according to the chromosomal positions. Chr04 contained the most *MaSPL* genes (11), while Chr11 only contained one (Supplemental Data Set S3). Next, a rootless phylogenetic tree was generated to elucidate the evolutionary relationships of *SPL* gene family members in banana, *Arabidopsis* and rice. All *SPL*s were clustered into nine subfamilies (Class I - IX), and *MaSPL*s were distributed in seven groups. Class I had the most members, including 12 *MaSPL*s, three *AtSPL*s and four *OsSPL*s, while both Class VII and VIII contained only two *AtSPL*s (Supplemental Fig. S3A).

**Fig. 2:**
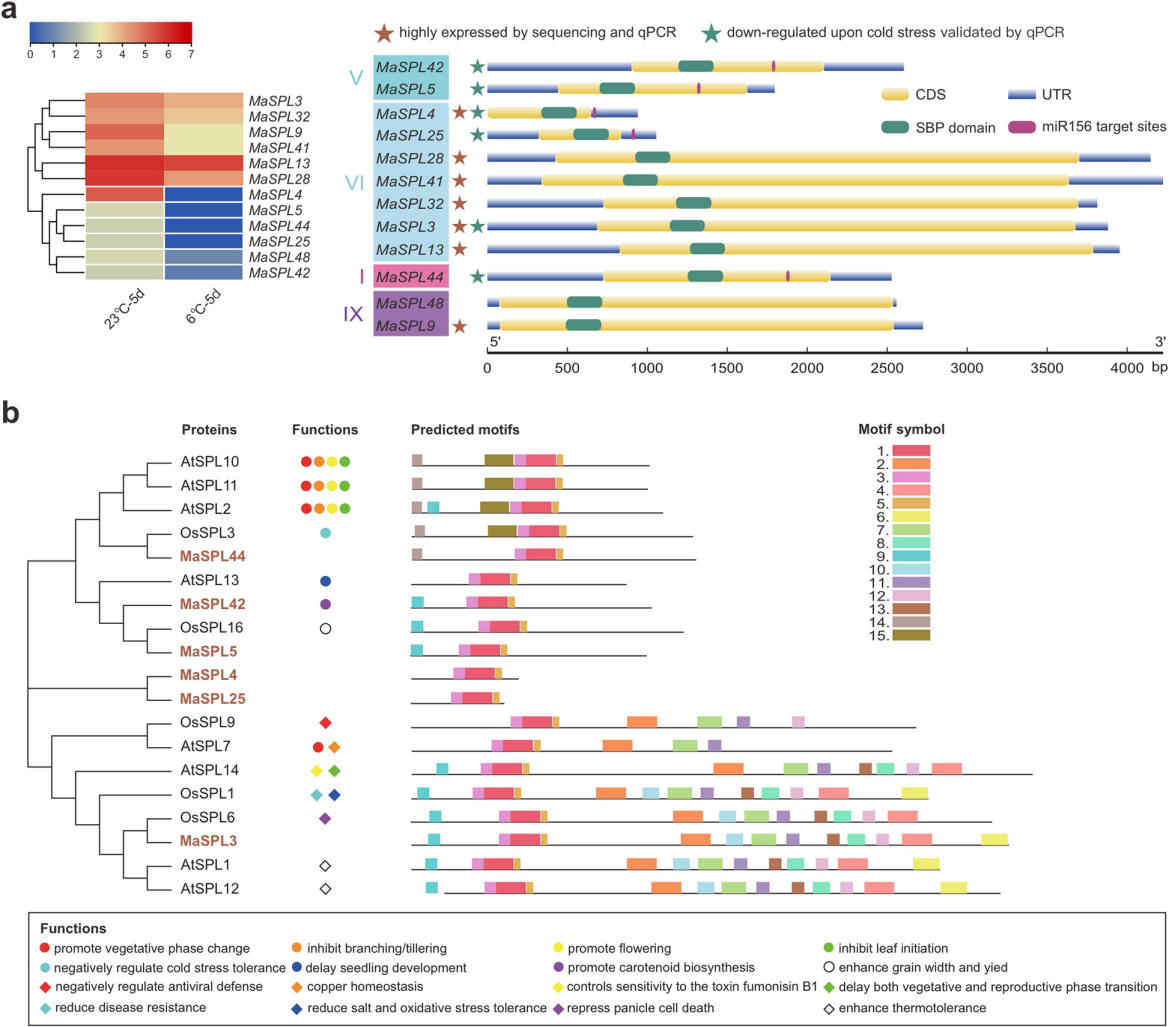
Phylogeny, structures and functions of expressed banana *SPL* family members.** a** Expression heat maps of 12 highly expressed *MaSPL*s under cold stress (left). Phylogenetic grouping and gene structure of expressed *MaSPL*s. Asterisks indicate highly expressed *MaSPL*s (brown) and down-regulated *MaSPL*s (green) under cold stress, respectively (right). The miR156 target sites are indicated with red boxes. **b** Protein motif arrangement, predicted stereostructure and evolutionary relationship of expressed *MaSPL*s and functionally characterized *SPL*s from *Arabidopsis* and rice. Phylogeny of these *SPL*s was determined based on their complete amino acid sequences by MEGA-X using the maximum-likelihood method. Bootstrap values were obtained using 1000 bootstrap replicates and shown next to the branches if > 50%. Three-dimensional structure of SPL proteins was predicted using SWISS-MODEL webserver. MEME motif search was based on the full-length protein sequences of SPLs. Conserved motifs are indicated by numbered colored boxes. Motif 1 and 3 indicate the two zinc fingers of the SBP domain. At, *A. thaliana*; Os, *Oryza sativa*; Ma, *Musa acuminata*

Our RNA-seq data showed that nearly 80% of the *MaSPL*s were not expressed or expressed at a very low level, and almost all expressed *MaSPL*s were significantly down-regulated by low temperature (Fig. 2a, Supplemental Fig. S4 and Supplemental Data Set S3). We then tested a group of highly expressed *MaSPL*s and further confirmed the downregulation of *MaSPL3/4/5/25/42/44* upon cold stress, which was similar to the expression pattern of miR528 as previously reported. Further bioinformatics analysis found that these highly-expressed and cold-repressed *MaSPL*s were distributed in subfamilies I, V, VI and IX. They all contained a highly conserved core SBP domain of 76 amino acids, but their coding and untranslated regions were highly variable (Fig. 2a). Five of these six *MaSPL*s were predicted to have miR156 target sites on their transcripts, among which three were located in the coding sequences near the C-terminal while the other two were in the 3’UTRs (Fig. 2a). Next, a phylogenetic categorization was performed for the six *MaSPL*s (*MaSPL3/4/5/25/42/44*) and *Arabidopsis*/rice *SPL* genes whose functions have been previously reported (Fig. 2b). It was further confirmed that the SBP domain with two zinc binding sites and a nuclear localization signal (NLS) overlapping with the second zinc finger were present in all SPL proteins of these three species. Similarly, little conservation was observed outside the SBP domain. Although some motifs were found conserved among subsets of these SPLs, their exact functions were largely unresolved (Fig. 2b). Overall, the tree was divided into two distinct groups based on the protein length. Most of the shorter SPL proteins lacking a large number of C-terminal motifs, most have been reported to regulate plant growth and development, while the other group of longer SPL proteins have been reported to regulate plant stress resistance (Fig. 2b). Five of the six banana SPL proteins were shorter and only retained a few motifs other than the conserved SBP domain, while MaSPL3 that was longer. MaSPL44 clustered with OsSPL3, which has been reported to negatively regulate cold stress tolerance (Zhou and Tang 2018). MaSPL5 and MaSPL42 were closely related to AtSPL13, which is involved in seedling development of *Arabidopsis*, and OsSPL16 that regulates grain width and yield of rice (Wang et al. 2012), while MaSPL42 has been reported to promote carotenoid synthesis in banana (Zhu et al. 2019a). MaSPL3 clustered with OsSPL6 that controls rice panicle cell death (Wang et al. 2018). However, the remaining MaSPL4 and MaSPL25 clustered together, appearing to have evolved independently, and their functions have not yet been referenced (Fig. 2b).

### Multiple MaSPLs specifically activate the transcription of *MIR528* gene

**Fig. 3:**
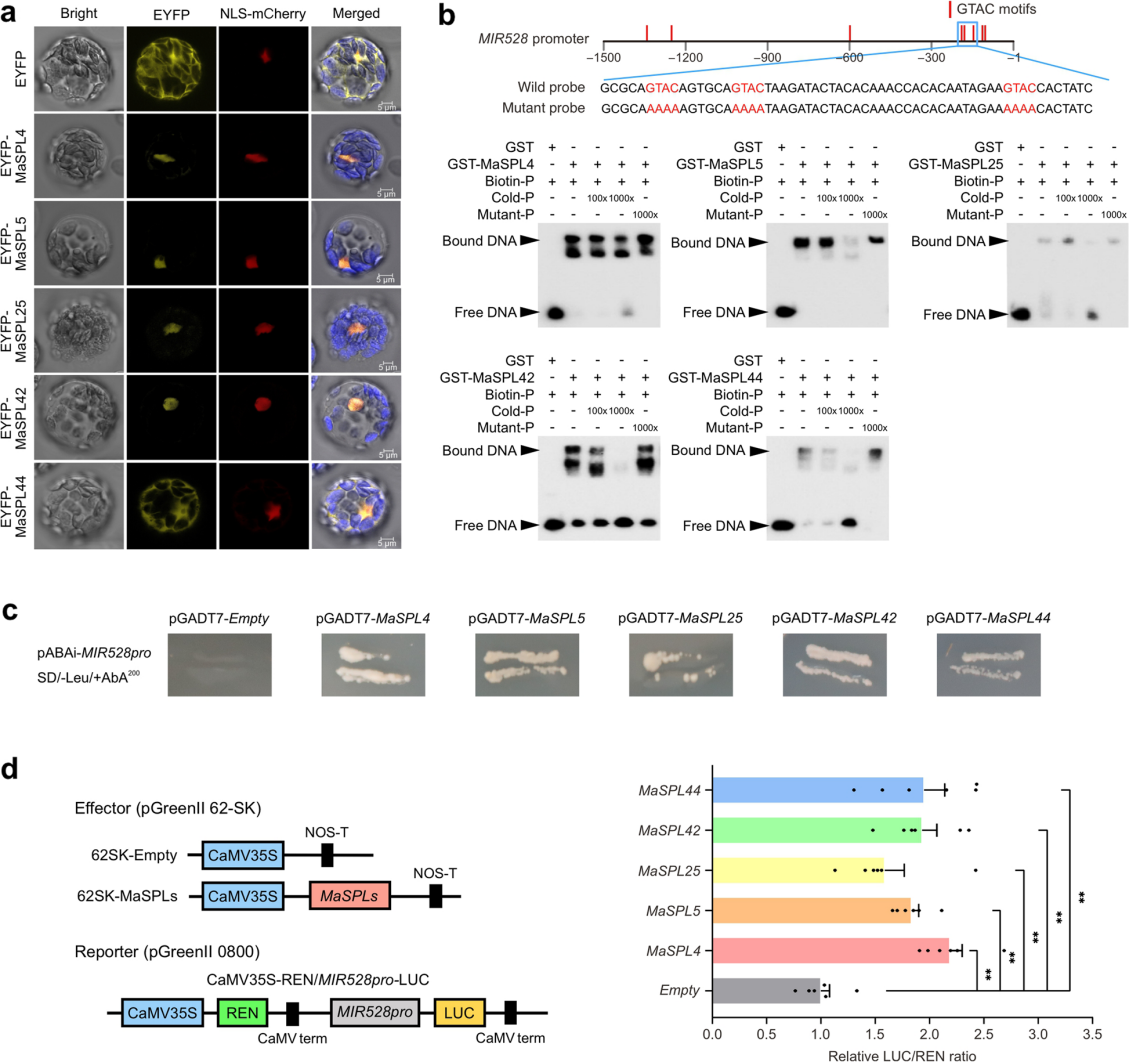
MaSPL4/5/25/42/44 positively control *MIR528* transcription by binding to the GTAC motifs in the *MIR528* promoter.** a** The subcellular localization of MaSPL4/5/25/42/44 in *Arabidopsis* mesophyll protoplasts, using NLS-mCherry as a nuclear localization marker. **b** EMSA of in vitro binding of MaSPL4/5/25/42/44 to the *MIR528* promoter. At the top is the *MIR528* promoter diagram, which contains eight GTAC motifs. Numbers below the diagram indicate the distance away from the *pre-miR528*. A biotinylated probe containing three GTAC motifs sequence were incubated with the GST-MaSPLs, while the probe incubated with GST protein was used as a negative control. Biotin-free probes containing three GTAC and AAAA motifs were used as cold competitors and mutant competitors, respectively. **c** Y1H assay shows that MaSPL4/5/25/42/44 bind to the promoter of *MIR528 in vitro*. A 210 bp fragment containing the EMSA probe sequence was inserted into the pABAi vector. The full-length CDS of MaSPL4/5/25/42/44 were cloned into the pGADT7 vector and the empty vectors were used as the negative control. Yeast with pABAi vector and pGADT7 vector were cultured on SD/-Leu/+AbA^200^ medium. **d** The activation of *MIR528* by MaSPL4/5/25/42/44 as determined by DLR. The LUC/REN ratio of the empty vector plus promoter-reporter was set as 1. Values are presented as means ± standard error (SE) of six biological replicates (*n* = 6). The asterisks (**) indicate significant differences at 0.01 level according to two-tailed unpaired Student’s *t*-test

To investigate whether these *MaSPL*s can bind to *MIR528* gene and regulate its expression in banana, we first examined their subcellular localization in vivo. Fluorescence microscopy showed that MaSPL4/5/25/42 were exclusively localized in the nucleus, while MaSPL44 was in both the nucleus and cytoplasm (Fig. 3a).

To further confirm that MaSPL4/5/25/42/44 could bind to the promoter of the *MIR528* gene in vivo, an electrophoretic mobility shift assay (EMSA) was performed, to further verify whether MaSPL4/5/25/42/44 can specifically bind to GTAC motifs on the *MIR528* promoter in vitro. To this end, the full coding sequences of MaSPL4/5/25/42/44 were fused with glutathione S-transferase (GST), and the GST-MaSPL fusion proteins were expressed in *E. coli* Rosetta (DE3) strain and purified. As shown in Fig. 3b, the purified recombinant proteins could bind to the *MIR528* promoter fragments containing GTAC motifs and induced obvious band mobility shifts. Further, the observed reduction of the band shift was due to the addition of increased unlabeled probes with identical sequences, rather than the mutated competitors (Fig. 3b). Next, in the yeast one-hybrid (Y1H) validation assay, when the AD vectors expressing *MaSPL4/5/25/42/44* were transferred to the recombinant yeast, the yeast could grow on the SD/-Leu/+AbA^200^ plate while the empty AD vector could not, suggesting the in vitro binding of MaSPL4/5/25/42/44 to the promoter of *MIR528* (Fig. 3c). Finally, a dual-luciferase reporter system (DLR) in tobacco leaves was performed. Compared with negative empty vector, vectors fused with *MaSPL4/5/25/42/44* significantly activated the transcription of reporter gene *LUC*, resulting in a significant increase in the LUC/REN ratio (Fig. 3d). Taken together, Y1H and EMSA assays validated in vitro that MaSPL4/5/25/42/44 could bind to the GTAC motif in the *MIR528* promoter region, while DLR experiment proved the activation of *MIR528* promoter by MaSPL4/5/25/42/44.

### The miR156/SPL module is involved in the chilling response of banana

In plants, a subset of *SPL*s are conserved targets of miR156 and the miR156/SPL module plays an important role in regulating plant fitness. To identify which of the above-mentioned MaSPLs that activate miR528 expression could potentially be targeted by miR156, we first performed a target prediction analysis for all *MaSPLs*. Somewhat unexpectedly, as many as 40 (~ 69%) of the total 58 *MaSPL*s contained a miR156 target site, among which the *MaSPL26* transcript had two target sites. These miR156-targeted *MaSPL*s distributed in subfamily I, II, III, V and a subset of subfamily VI. The miR156 target sites in the VI subfamily were located at the 3’UTR, while those in the other subfamilies were located in the CDS region (Supplemental Fig. S3B). Moreover, *MaSPL4/5/25/42/44*, which have been shown to activate the miR528-PPO module, were all predicted as targets of miR156 (Fig. 2a, Supplemental Fig. S3B).

**Fig. 4:**
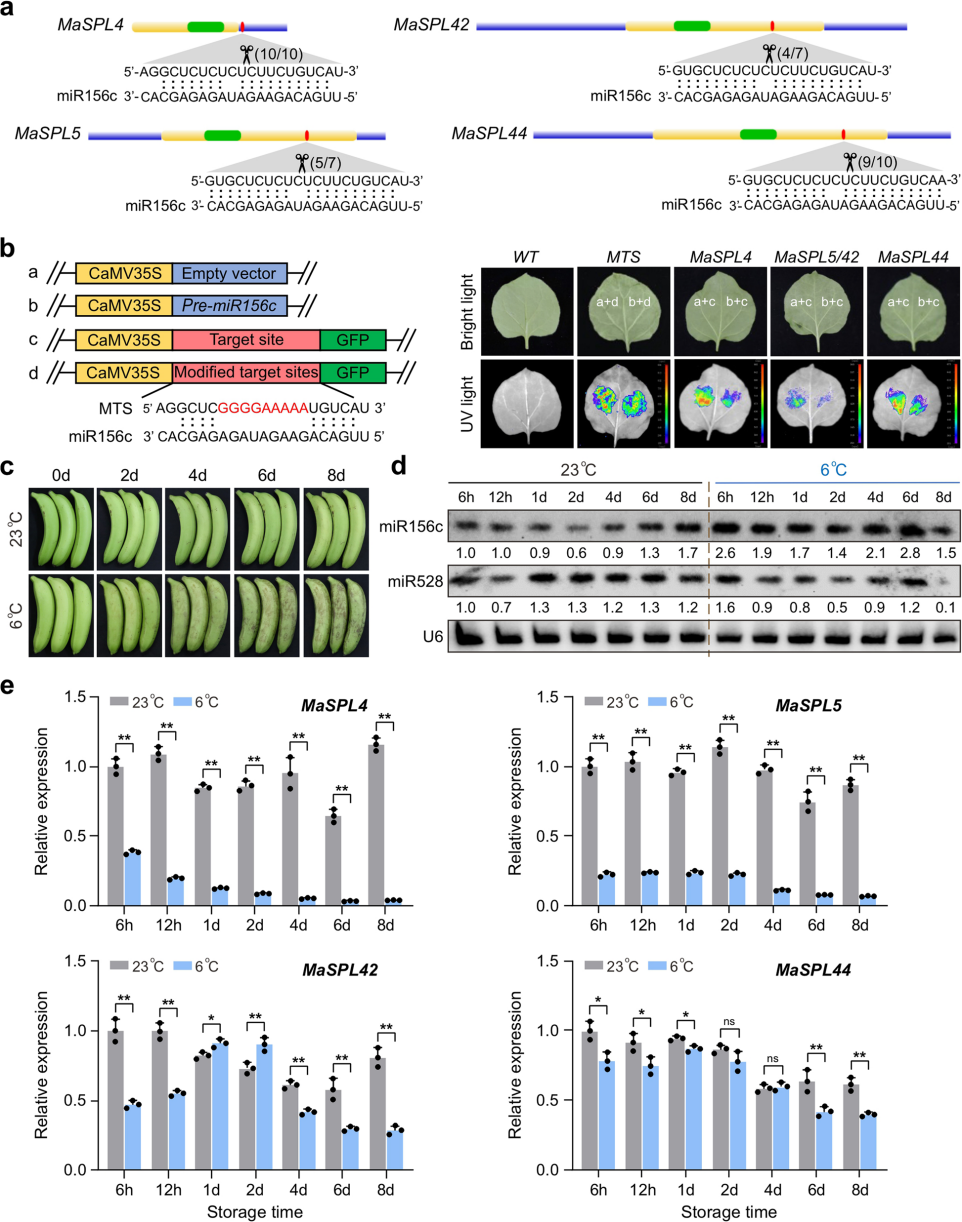
Validation and expression analysis of miR156-MaSPL module in banana peel under cold stress.** a** Alignments of miR156 with their targeted *MaSPL*s validated in banana. Scissors indicate the cleavage sites of the targeted *MaSPL4/5/42/44*, as detected by RLM-5’-RACE assay and the factions next to the scissors show the numbers of clones with an identified 5’ end detected in the total sequenced clones. **b **In vivo targeting validation of miR156-targeted *MaSPL*s. Four overexpression vectors were constructed for the transient expression assay in tobacco. a: empty vector; b: vector overexpressing *pre-miR156c*; c: vector overexpressing GFP fused with miR156 target sites of *MaSPL4/5/42/44*; d: vector overexpressing GFP fused with miR156 modified target sites of *MaSPL4/5/42/44*. Co-infiltrated leaves and control leaves were photographed on the 3rd day after infiltration under bright light and UV light. Combinations of vectors used in the assay were shown on the leaves. **c** A time-course phenotype of banana fruit under cold stress. **d** Detection of miR156c and miR528 levels in banana peel during cold stress. U6 was probed as a loading control. Numbers below the bands are the mean gray values of the samples relative to those of U6, calculated by the Image J software. The control (23℃) sample at 6 h was set as 1. **e** Gene expression pattern of miR156-targeted *MaSPLs* in banana peel during cold stress. The *RPS2* gene was used as an internal reference. Data are presented as the means ± SD of three biological replicates (*n* = 3) and significant differences are indicated with asterisks, as determined by two-tailed unpaired Student’s *t*-test (*, *P* < 0.05; **, *P* < 0.01, ns, not significant)

To validate miR156c targeting of *MaSPL4/5/25/42/44*, both RLM-5’-RACE and transient co-expression assays in tobacco leaves were conducted. The 5’-RACE experiment showed that the cleavage sites of miR156c on *MaSPL4* and *MaSPL44* were located between the 10th and 11th nucleotides of miR156, while on *MaSPL5* and *MaSPL42*, between the 11th and 12th nucleotides (Fig. 4a). However, no cleavage was detected on *MaSPL25* (data not shown). Transient co-expression assays in tobacco leaves were performed to further confirm the targeting of *MaSPL4/5/42/44* by miR156c in vivo. Two vectors were used in this experiment, where one was used to overexpress miR156c and the other was used to insert either a target site or a modified target site before GFP. Vectors were combined in pairs and introduced into *Agrobacterium*. It was observed that three days after infiltrating into tobacco leaves, the green fluorescence signal weakened where miR156c and its target site were co-expressed (b + c), while the signals for other combinations (a + c, a + d and b + d) remained largely unchanged (Fig. 4b). These results provided evidence that miR156c interacted with and suppressed the target sites in *MaSPL4/5/42/44*, leading to the inhibition of GFP expression. To further verify the involvement of miR156/SPL module in banana, we tested banana transverse slices at room temperature where miR156c was either overexpressed (OE-miR156c) or silenced (STTM-miR156c). Six days after infiltration, overexpression of miR156c promoted the browning of banana slices while silencing miR156c delayed the browning, compared to the empty vector control (Supplemental Fig. S5A). We then measured both miR156c and *MaSPL4/5/42/44* expression levels in the corresponding samples, and the miR156c abundance increased by 0.6 times in the OE-miR156c samples but reduced by 50% in the STTM-miR156c samples, compared to the control (Supplemental Fig. S5B). Correspondingly, *MaSPL4/5/42/44* and miR528 were down-regulated in the OE-miR156c samples but up-regulated in the STTM-miR156c samples (Supplemental Fig. S5C). Those results further proved that *MaSPL4/5/42/44* were the authentic targets of miR156c.

Next, we profiled the expression patterns of miR156c-*MaSPL4/5/42/44* under cold stress of banana fruit during a time-course. As shown in Fig. 4c, with the continuation of cold stress, obvious browning symptom was observed in banana peel. Based on our previous sRNA-seq data, there were twelve miR156 family members in banana, among which miR156c had a relatively high abundance (Zhu et al. 2019). Hence, we further tested the accumulation of miR156c as well as miR528 by RNA blot. It was found that the expression of miR156c was significantly induced as early as 6 h under cold stress, and this induction was sustained until 6 d (Fig. 4d). Conversely, the expression of miR528 was reduced by cold, especially from 1 d onward. Accordingly, quantitative real-time polymerase chain reaction (qRT–PCR) results showed that the validated miR156c-targeted *MaSPL4/5/42/44* that can activate the miR528-PPO module were all down-regulated upon cold stress (Fig. 4e).

### The miR156c-MaSPL4 module regulates banana chilling response through the miR528-PPO module

**Fig. 5:**
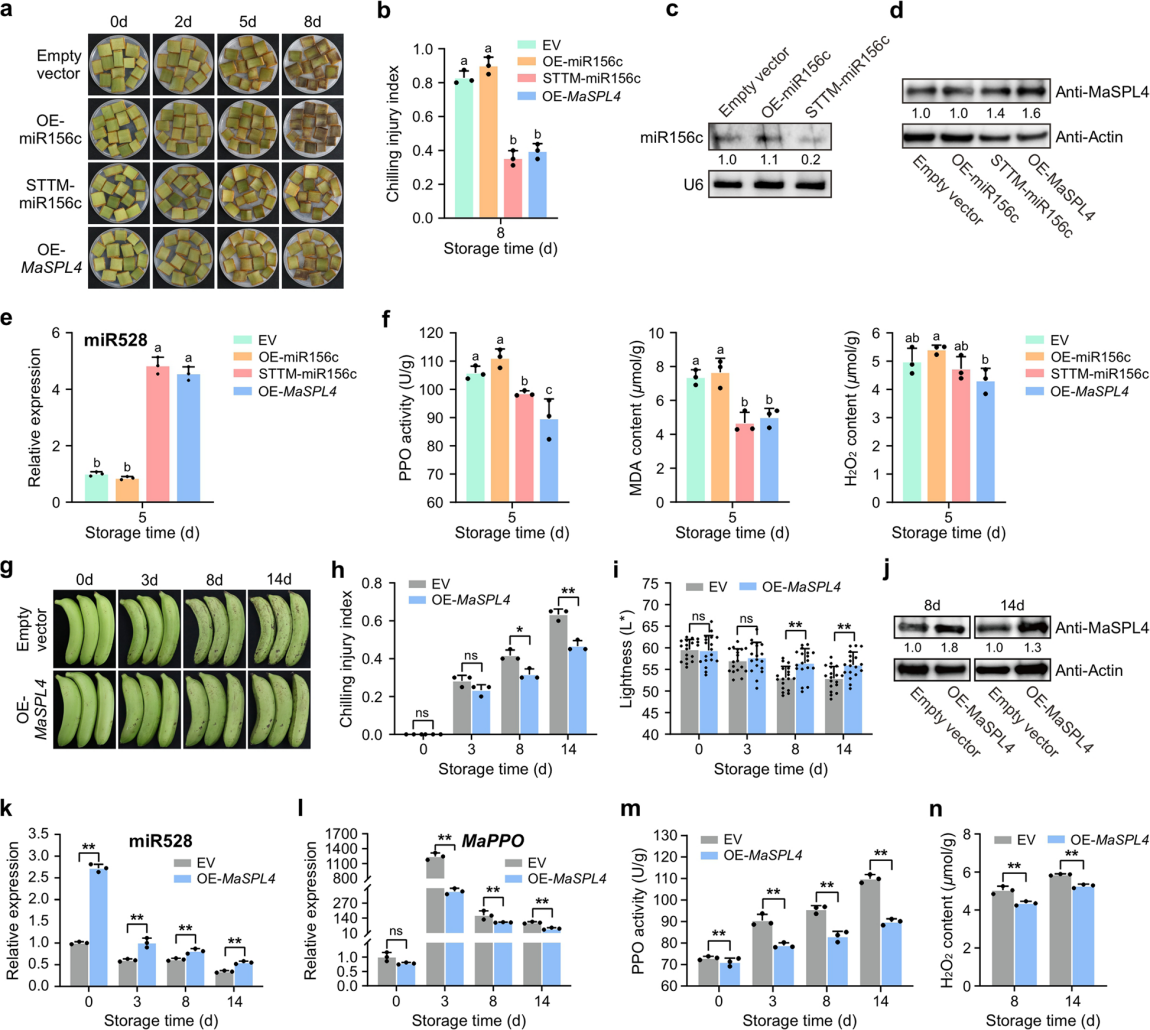
The miR156c-MaSPL4 regulates banana chilling response through the miR528-PPO module.** a** Phenotype and **b** chilling index of transient OE-miR156c, OE-*MaSPL4* and STTM-miR156 banana peel slices under cold stress. Data are means ± SD with three biological replicates (*n* = 3). **c** Abundance of miR156c in EV, OE-miR156c and STTM-miR156c samples on day 5 of cold stress. U6 was used as an internal reference. Numbers below the bands are the mean gray values of the samples relative to those of U6, calculated by the Image J software. The EV sample was set as 1. **d** MaSPL4 protein level in different samples on the fifth day was quantified by western blot, Actin was used as an internal reference. Numbers below the bands are the mean gray values of the samples relative to those of Actin, calculated by the Image J software. The EV sample was set as 1. **e** The miR528 abundance was quantified by RT-qPCR, using U6 as an internal reference. Data are means ± SD with three biological replicates (*n* = 3). **f** Changes in the PPO activity, MDA content and H_2_O_2_ level in control (empty vector) and transient transgenic samples. Error bars indicate the SD for three biological replicates (*n* = 3). **g** Changes in appearance, **h** chilling injury index and **i** lightness (L^*^) in OE-*MaSPL4* and control banana fruit under cold stress. Data are means ± SD with *n* = 3 or *n* = 18. **j** Quantification of MaSPL4 protein levels by western blot, Actin was used as an internal reference. Numbers below the bands are the mean gray values of the samples relative to those of Actin, calculated by the Image J software. The EV samples at 8 d and 14 d were set as 1. **k** The expression of miR528 and **l***MaPPO* quantified by RT-qPCR. U6 and *RPS2* were used as internal references, respectively. Data are means ± SD with *n* = 3. **m** Changes in PPO activity and **n** H_2_O_2_ level in OE-*MaSPL4* and control (empty vector) banana peel. Data are means ± SD with *n* = 3. The different letters indicate that the difference is significant at 0.05 level and asterisks indicate significant differences between control and transient transgenic samples at each time point according to one-way analysis of variance (ANOVA), followed by Duncan’s multiple‐comparison post hoc test **b**,** e**,** f** and two-tailed unpaired Student’s *t*-test **h**,** i**,** k**,** l**,** m**,** n**), respectively (*, *P* < 0.05; **, *P* < 0.01, ns, not significant)

Direct activation of miR528 by miR156c-targeted MaSPL4/5/42/44 supports a model that the miR156-SPL module acts through the miR528-PPO module to control banana chilling response. Therefore, we utilized a transient transformation system in the banana peel to further verify this model. Based on the facts that miR156c-targeted *MaSPL4* was highly expressed with most responsiveness to cold stress, and that MaSPL4 had the strongest effect on *MIR528* promoter binding and activation, *MaSPL4* was selected to perform subsequent functional verification. In a set of parallel experiments, miR156c was either overexpressed or STTM-silenced, and *MaSPL4* was overexpressed, with empty vectors as negative controls. After eight days of cold stress, the chilling injury symptom of banana peel slices with miR156c overexpression was more serious than that of the empty vector, while the chilling phenotype of the samples with silencing of miR156c and *MaSPL4* overexpression was less severe than that of the control, especially with the miR156c silencing group (Fig. 5a, b). The miR156c abundance increased in the OE-miR156c samples but reduced in the STTM-miR156c samples, compared with the control (Fig. 5c). Correspondingly, the protein accumulation of MaSPL4 and miR528 abundance were all enhanced to varying degrees in both OE-*MaSPL4* and miR156c-silenced banana peel slices but decreased in OE-miR156c samples, compared with empty vector (Fig. 5d, e). Changes in PPO activity, MDA level and H_2_O_2_ content were also determined. In general, STTM-miR156c and OE-*MaSPL4* samples had lower PPO enzyme activity, MDA and H_2_O_2_ content than OE-miR156c and EV samples, which resulted in less severe symptoms of cold injury in STTM-156c and OE-*MaSPL4* samples (Fig. 5f).

Additionally, *MaSPL4* was transiently overexpressed in banana whole fruit and the results were consistent with those observed in banana peel slices, where the symptom of chilling injury of banana fruit was delayed and alleviated after overexpression of *MaSPL4* (Fig. 5g). On 8 d and 14 d of cold stress, the chilling injury index of banana with *MaSPL4* overexpression was significantly lower with higher lightness compared to the empty vector (Fig. 5h, i). Correspondingly, the protein level of MaSPL4 in OE-*MaSPL4* banana was significantly higher than the control. The abundance of miR528 was significantly increased, and the expression of its target gene *MaPPO* was significantly down-regulated (Fig. 5j, k, l). Also, the activity of PPO enzyme and the H_2_O_2_ level were all significantly decreased in the OE-*MaSPL4* fruit, compared with the control (Fig. 5m, n). This chain of experimental evidence showed that miR156c could aggravate the chilling symptoms of banana under cold stress whereas MaSPL4 could alleviate it. Moreover, this miR156c-MaSPL4 regulatory module transmitted the cold stress signal through the miR528-PPO module to control the chilling response of banana fruit.

### MasplSPL4 potentially acts as a hub of chilling response hierarchy regulatory network in banana

The highly conserved C-repeat binding factor (CBF) pathway has been well characterized as correlated with cold response in plants (Shi et al. 2018). But it is also widely agreed that, in addition to CBF, at least a third of the low-temperature regulons have yet to be unveiled, including a variety of other transcription factors, most of which remain unknown for banana, an economically important species but highly sensitive to chilling injury. Based on the preliminary phenotypic and functional analysis of MaSPL4, we hypothesized that this transcription factor may be a key factor regulating cold stress response in banana. Therefore, a DAP-seq experiment was performed for MaSPL4, to try to identify more downstream pathways that might be regulated by it. In parallel, we did transcriptome profiling by RNA-seq on OE-*MaSPL4* and control fruit samples at 14 d when chilling injury symptoms were clearly observed, to identify differentially expressed genes (DEGs) involved in the cold response in banana.

**Fig. 6:**
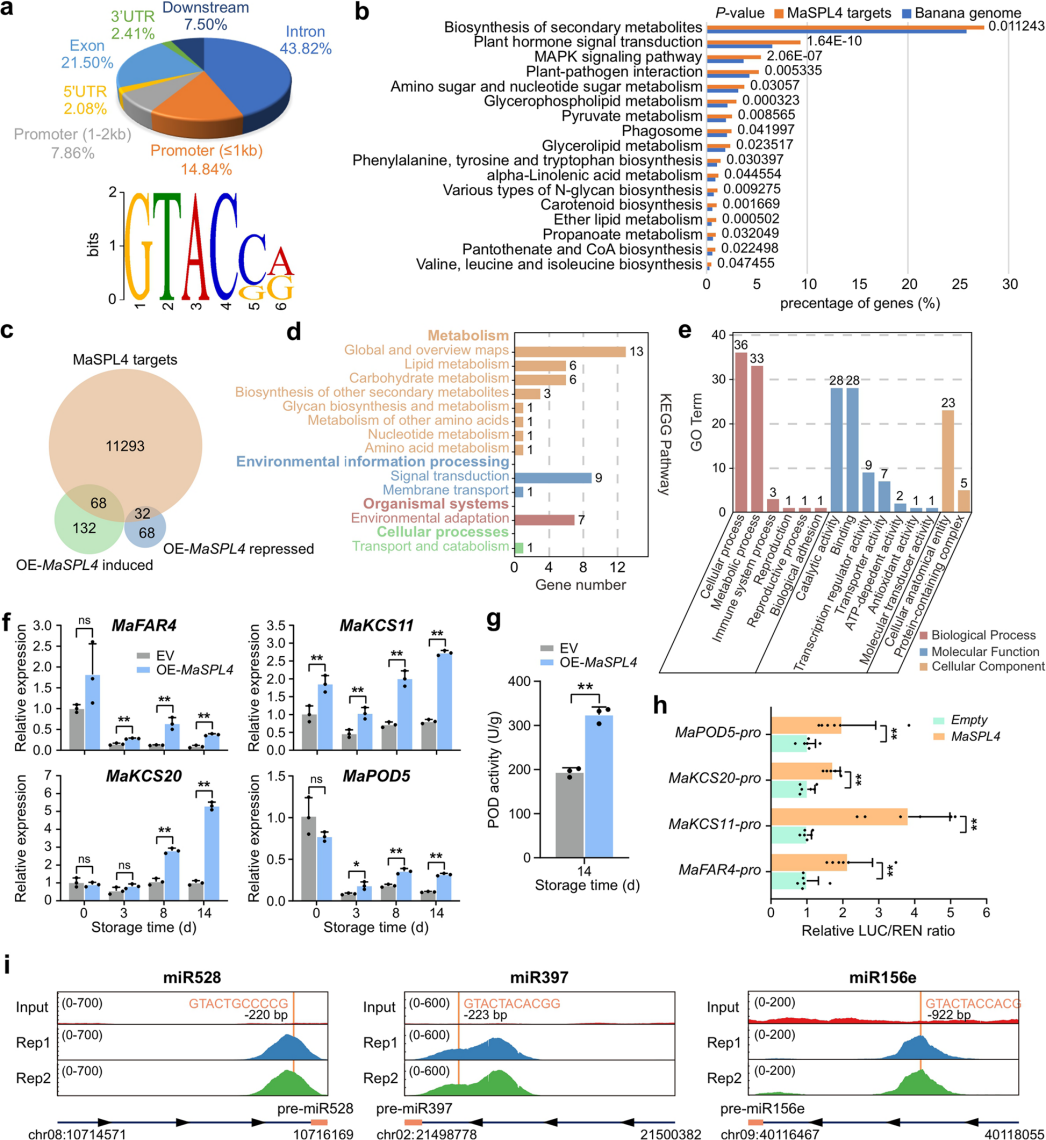
Target genes of MaSPL4 identified via combined genome-wide DAP-seq and RNA-seq analyses.** a** Distribution of the MaSPL4-binding peaks and the putative DNA-binding motif of MaSPL4 identified by DAP-seq in the promoter region of the target genes. **b** KEGG categorization of MaSPL4-binding genes. Enriched KEGG pathways of MaSPL4 compared with all *Musa acuminata* genes are shown. A false discovery rate (FDR) cut-off was set on the basis of a *P* < 0.05. **c** Venn diagram displaying the numbers of overlapping genes among MaSPL4 target genes (orange circle) revealed by DAP-seq and differentially expressed genes (DEGs, green and purple circles) identified by RNA-seq in OE-*MaSPL4* samples. DEGs refer to those differentially expressed after 14 days of cold treatment when fruit showed obvious chilling injury. **d** Enriched KEGG pathways and **e** GO terms of 68 OE-*MaSPL4* induced targets. **f** Expression analysis of selected MaSPL4 target genes related to lipid metabolism and REDOX, respectively. The *RPS2* gene was used as an internal reference. **g** Changes in POD activity in OE-*MaSPL4* and control (empty vector) banana peel. Error bars indicate the SD of three biological replicates (*n* = 3) and asterisks indicate significant differences between control and transient transgenic samples at each time point, according to two-tailed unpaired Student’s *t*-test (*, *P* < 0.05; **, *P* < 0.01, ns, not significant). **h** DLR assay showing the activation of selected lipid metabolism and REDOX-related genes by MaSPL4. The LUC/REN ratio of the empty vector plus promoter-reporter was set as 1. Values are presented as means ± SD (*n* = 6). The asterisks (**) indicate significant differences at 0.01 level according to two-tailed unpaired Student’s *t*-test. **i** Binding peaks of MaSPL4 in the promoter of *MIR528*, *MIR397* and *MIR156e* identified by DAP-seq. Both miRNA precursor and upstream 1.5 kb regions are shown. The arrows indicate the direction of *MIRNA* gene transcription and the solid orange lines indicate the positions of MaSPL4 binding motif

Up to 55,990 peaks were detected via DAP-Seq (Supplemental Data Set S4) and it was shown that approximately 22.7% of the MaSPL4 binding sites were enriched in the gene promoter regions, including 14.84% of the peaks within 1 kb upstream of the transcription start site (TSS), and 7.86% within 1–2 kb upstream TSS (Fig. 6a). *De novo* motif analysis of MaSPL4 DAP-seq peaks identified GTAC(C/G)(A/G) as the most representative motif enriched within the MaSPL4 binding regions (Fig. 6a, Supplemental Data Set S5). Further, 11,393 genes with DAP-peak-containing promoter regions were identified as MaSPL4 target genes (Supplemental Data Set S6). Kyoto Encyclopedia of Genes and Genomes (KEGG) enrichment analysis of these putative MaSPL4 target genes uncovered that MaSPL4 participates in multiple pathways, including biosynthesis of secondary metabolites, hormone signal transduction, MAPK signaling pathway, plant-pathogen interaction, etc. (Fig. 6b). Further, RNA-seq identified a total of 300 DEGs in OE-*MaSPL4* samples compared to control, among which 200 DEGs were up-regulated while 100 were down-regulated under cold stress. The complete DEG list screened by pairwise comparison is provided in Supplemental Data Set S7. Next, combing RNA-seq with DAP-seq data, a total of 100 MaSPL4-binding DEGs were identified, among which 68 and 32 were induced and repressed in the overexpression samples, respectively (Fig. 6c, Supplemental Data Set S8). KEGG and GO enrichment analyses revealed that these OE-*MaSPL4*-induced targets were significantly associated with lipid metabolism and antioxidation-involved environmental adaptation (Fig. 6d, e). Genes related to lipid metabolism included those in cutin synthesis (*MaFAR4*) and fatty acid elongation (*MaKCS11* and *MaKCS20*), while the redox-related gene included that in phenol oxidation (*MaPOD5*). Then, a group of up-regulated target DEGs involved in lipid metabolism and REDOX were selected to further confirm their regulation by MaSPL4. Gene expression analyses confirmed that these target genes were mostly inhibited by cold stress in the control fruit, while they were enhanced to varying degrees in the OE-*MaSPL4* banana fruit (Fig. 6f). The enzymatic activity of peroxidase (POD) coded by the MaSPL4-targeted *MaPOD5* was also measured, and it was found that its activity in the OE-*MaSPL4* samples was higher than that in the control. This suggested that overexpression of *MaSPL4* might have gradually improved the ability of fruit to clear excessive reactive oxygen species (ROS) at a later stage (Fig. 6g). To verify *MaFAR4*, *MaKCS11*, *MaKCS20* and *MaPOD5* as target genes of MaSPL4, DLR experiments were conducted and the results confirmed that these genes could be activated by MaSPL4 (Fig. 6h).

In addition to the above-mentioned MaSPL4-binding targets related to lipid metabolism and REDOX, it was also revealed that a group of miRNAs could be potentially under the transcriptional regulation of MaSPL4, demonstrating MaSPL4 as a key hub gene intertwining the miRNA regulatory network in banana (Fig. 6i, Supplemental Fig. S6). The binding site of MaSPL4 in the promoter of *MIR528* gene was confirmed, and the DAP-seq data also revealed MaSPL4 binding of another Cu-miRNA, miR397, which has been widely reported to be involved in plant stress response (Supplemental Fig. S6) (Pilon 2016). Moreover, MaSPL4 could bind the promoter of *MIR156e*, forming a feedback regulatory loop for the miR156-MaSPL4 module homeostasis (Fig. 6i).

## Discussion

**Fig. 7:**
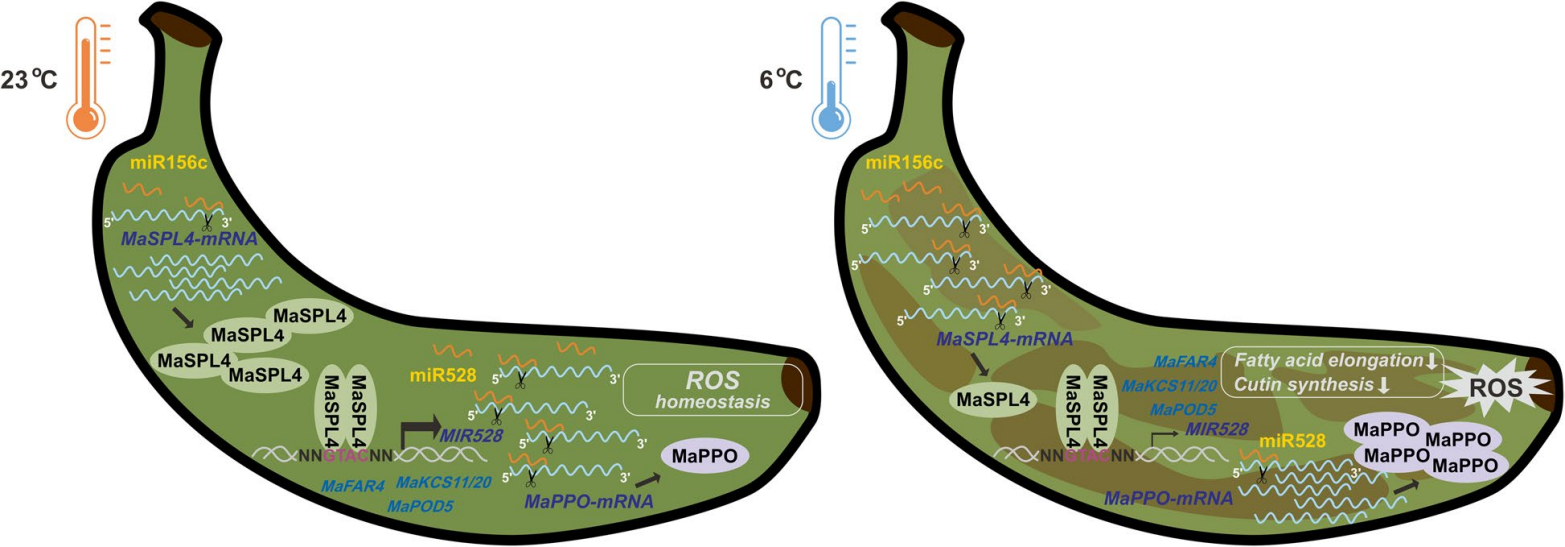
A working model for miR156c-MaSPL4-miR528-PPO regulatory cascade in banana chilling response. This study demonstrates a MaSPL4-linked cascade mechanism underlying banana chilling injury. miR156c recognizes and cleaves the transcript of *MaSPL4*, negatively regulating its expression. MaSPL4 forms homologous dimers for self-activation and binds the promoters of *MIR528* to activate its expression, while miR528 targets and suppresses *MaPPO* expression and downstream ROS perturbation. Under normal temperature, the ROS homeostasis is maintained with a higher *MaSPL4* expression resulted from marginally expressed miR156c, and a lower MaPPO activity by miR528 inhibition. Under cold stress, miR156c accumulates and causes a significant reduction of MaSPL4 and miR528 expression, leading to an enhanced *MaPPO* expression, and thus ROS burst and chilling injury as mainly manifested by the peel browning. In addition to miR528, MaSPL4 also potentially targets a group of genes involved in lipid metabolism and antioxidant capacity, suggesting that MaSPL4 may act as a hub of chilling response independently from the CBF regulon. Hence, the up-regulation of MaSPL4 may contribute to the higher cold tolerance of OE-*MaSPL4* banana samples

Unearthing key factors and regulatory mechanisms of plant response to cold stress will help the future design of effective strategies to improve cold tolerance of important crop species, including banana. This study reveals a MaSPL4-linked cascade mechanism underlying banana chilling injury. In particular, a hierarchical network of interactions between protein-coding transcription factors and non-coding RNAs involved in the regulation of chilling injury is partially uncovered (Fig. 7).

Since firstly identified in snapdragons (Klein et al. 1996), a large number of *SPL* gene family members have been characterized in various plant species. So far, 16, 19, 31, and 13 *SPL* genes have been reported in *Arabidopsis*, rice, maize, and moss (*Physcomitrella patens*), respectively (Arazi et al. 2005; Cardon et al. 1999; Hultquist and Dorweiler 2008; Miura et al. 2010; Riese et al. 2008). It is also worth noting that a subset of *SPL* transcripts has evolved to be conservatively targeted by miR156 for cleavage and/or translational repression. For example, 10 out of 16 *Arabidopsis* *SPL*s are miR156 targets (Wu et al. 2006), and 11 out of 19 *SPL*s in rice are targeted by miR156 (Xie et al. 2006). Similarly in this study, 58 members of the *SPL* gene family were identified in banana and 40 of them were potentially targeted by miR156 (Supplemental Fig. S3B). This highly conserved and rigorous targeting relationship suggests important roles of miR156-SPL module in regulating plant life.

In plants, miR156 and its target *SPL* genes have been well studied for growth and development, including leaf development, architecture, juvenile-to-adult phase change, flowering, fertility, and fruit ripening (Jiao et al. 2010; Schwarz et al. 2008; Wang and Wang 2015; Wang et al. 2009; Wu et al. 2009; Xing et al. 2010). Moreover, the function of the miR156-SPL module in regulating plant response to various stresses like temperature stress has also been recently reported. It has been shown that miR156 is induced by heat stress and plays an important role in the stress memory (Stief et al. 2014). Overexpression of OsmiR156k reduces cold stress tolerance of rice by down-regulating *SPL3*, *SPL14* and *SPL17* (Cui et al. 2015). Similarly, Mdm-miRNA156 has been reported as a negative regulator of cold tolerance in apple (Shen et al. 2023). In the present study, we found that banana miR156c was induced with significant down-regulation of its target genes *MaSPL4/5/42/44* (Fig. 4d, e) at the early stage of cold stress, indicating the potential role of miR156-SPLs in cold response of banana.

miRNAs are encoded by *MIRNA* genes which are mostly located in the intergenic region as independent transcription units in plants, and a few others are located in the protein-coding genes and co-transcribed with host genes (Rajagopalan et al. 2006; Yan et al. 2012; Yang et al. 2012). A considerable number of regulators have been reported to cooperate with RNA polymerase II (Pol II) and synergistically participate in *MIRNA* gene transcription (Achkar et al. 2016; He et al. 2022). In addition, common transcription factors can also regulate the transcription of *MIRNA* genes. For example, the *Arabidopsis* SPL9 and SPL10, which are targeted by miR156, can both promote *MIR172* transcription. These two SPLs can also positively regulate *MIR156* transcription, thus forming a negative feedback regulatory network to maintain the stable expression of miR156 (Wu et al. 2006, 2009). A subsequent study revealed that the APETALA2 (AP2) transcription factor can promote *MIR156e* transcription and inhibit *MIR172b* transcription (Yant et al. 2010). Also, FUSCA3 and POWERDRESS promote the transcription of *MIR156* and *MIR172*, respectively (Wang and Perry 2013; Yumul et al. 2013). It has been demonstrated that the establishment of leaf polarity in *Arabidopsis* involves the repression of *MIR165/166* by class III and class II HD-ZIPs adaxially, while a feedback repression of class III HD-ZIPs by miR165/166 abaxially (Merelo et al. 2016). In rice, among the 19 members of the *SPL* gene family, only OsSPL9 can regulate *MIR528* transcription for the orchestration of antiviral response (Yao et al. 2019). Our previous study reveals that the down-regulated expression of miR528 under cold stress is a key factor leading to the ROS imbalance and chilling injury in banana (Zhu et al. 2020). In this study, multiple SPL binding sites were found in the *MIR528* promoter of banana (Supplemental Fig. S2) and several MaSPLs in banana, i.e., MaSPL4/5/25/42/44, were able to bind to the *MIR528* promoter and activate its expression (Fig. 3), which was unlike the situation in rice. Another difference was that *OsSPL9* is not a target gene of miR156, whereas *MaSPL4/5/42/44* that could regulate *MIR528* were all target genes of miR156 (Fig. 4a, b). These differences suggest that the regulatory specificity of banana SPLs to downstream miR528 is not as strong as that of rice, which may also be due to the influence of upstream miR156 targeting.

In addition to miR528, the combined DAP-seq and RNA-seq data also identified several cold-related genes involved in lipid metabolism and antioxidant activity, which were directly regulated by MaSPL4 (Fig. 6). The expression of these genes was consistently down-regulated by cold stress in the control fruit but significantly enhanced in the OE-*MaSPL4* banana fruit, suggesting that MaSPL4 could enhance the cold tolerance in banana through these pathways. Very long chain fatty acids (VLCFAs) are fatty acids with a chain-length of ≥ 20 carbons, serving as the basis of various lipids that regulate plant stress responses, and 3-ketoacyl-CoA synthetase (KCS) is a key rate-limiting enzyme that determines the final chain length and content of VLCFAs (Gong et al. 2023). It has been reported that AtKCS1 can enhance VLCFA contents and chilling tolerance in *Arabidopsis* (Chen et al. 2020), and GhKCS13 is an important player in resisting cold stress by modulating lipid and oxylipin biosynthesis in cotton (Wang et al. 2020). On the other hand, there is ample evidence that low temperatures can induce oxidative stress through ROS production in plants, and excessive ROS affect plant life by destroying many important cellular components such as DNA, lipids and proteins. Accordingly, plants have evolved enzymatic detoxification mechanisms to scavenge ROS, including superoxide dismutase (SOD), peroxidase (POD), catalase (CAT) and other enzymes. Among them, POD reduces hydrogen peroxide to water by energy transfer from active peroxide to glutathione. Recently, a study demonstrates a MaMAPK3-MaICE1-MaPOD P7 pathway that positively improves the cold tolerance in banana (Gao et al. 2021). In the present study, banana homologs of *KCS* (*MaKCS11/20*), *POD* (*MaPOD5*) genes were all up-regulated by the overexpression of *MaSPL4* (Fig. 6f), with POD activity also elevated. These results corresponded well with the enhanced cold tolerance of banana (Fig. 5d).

In this study, we used transient overexpression of miR156c and STTM method to silence miR156c to verify the function of miR156c under cold stress. It was shown that miR156c could negatively regulate cold tolerance in banana (Fig. 5a, b, c), which was consistent with previous reports on its homologs miR156k in rice and miR156 in apple (Cui et al. 2015; Shen et al. 2023). Accordingly, the target gene of miR156c, *MaSPL4*, positively regulated cold tolerance (Fig. 5). These findings confirmed the regulatory role of the miR156c-MaSPL4 module in cold tolerance of banana. Taken together, our data argue for the existence of a novel cold stress pathway integrated by the miR156-targeted MaSPL4 in banana, in addition to the well-known CBF regulon. This miR156c-MaSPL4 regulatory module mediates banana chilling response via the miR528-PPO module and multiple other pathways, which provides new targets for breeding strategies aiming to enhance banana tolerance and to improve fruit quality in an environment below 13℃ (Fig. 7).

## Materials and methods

### Plant material and low temperature stress treatments

Banana fruit (*Musa acuminata* AAA group, Cavendish subgroup) at 70–80% maturation were harvested from an orchard in Guangzhou, China. Fruit with uniformity size and color as well as non-polar mechanical damage and spots were selected and then soaked in 0.05% (w/v) Sporgon solution for 3 min to remove potential microorganisms and air-dried. Two temperatures were set in this study: control at 23 °C and cold stress temperature at 6 °C. For each temperature treatment, 90 fruit fingers were selected, packed in 0.02-mm-thick polyethylene bags, and divided into three groups of 30 fingers each as biological replicates. For each replicate sampling, peel tissues were collected at 2, 4, 6, and 8 d from the middle part of three fruit fingers, immediately frozen in liquid nitrogen, and stored at -80 °C until use. Sample at 0 d was used as the starting point (before treatment) and shared by treatments.

### Transient expression in banana peel slice and fruit

A binary vector, pRHV, which contains an Ubi promoter was used in this work to overexpress or silence genes (Supplemental Fig. S1). The sequences of *MIR528* and *MIR156c* contain the *pre-miR528* and *pre-mi156c*, and the CDS of *MaSPL4*, *STTM528* and *STTM156c* modules were cloned into the *Bam*H I/*Hind* III sites of pRHV between the Ubi promoter and the Nos terminator. The recombinant vectors and the empty vector pRHV were transformed into the *Agrobacterium* strain EHA105 which were used for transient gene expression studies in the banana peel. Banana fruits were soaked in 1% sodium hypochlorite solution for 3 min and cut the banana peel into cubes. The cubes were immersed in *Agrobacterium* solution and performed vacuum (− 70 kPa) infiltration. The vacuum was released slowly to assist the bacterial invasion of the fresh tissue. After vacuum infiltration, peel cubes were rinsed three times with ultra-purified water and cultured on Murashige and Skoog (MS) medium at 25℃ for 2 days. It was then stored at 6℃ for low-temperature stress treatment and peel cube samples were collected at 0, 2, 5, 8 d, immediately frozen in liquid nitrogen, and stored at -80 °C until use. Transient expression experiments using 50 fruit fingers. For banana whole fruit transient expression, 1.0 ml *Agrobacterium* strain EHA105 carrying pRHV-MaSPL4 and empty vector were injected into banana fruit. After infection with the pRHV-MaSPL4 and empty vector, the fruits were stored at room temperature for two days and transferred to 6℃ for 14d. The fruit chilling index and color index were determined on days 0, 3, 8 and 14d (under 6℃). And the peel samples were collected at the same time.

### Determination of physiological indexes

Chilling injury (CI) index, fruit peel color and malondialdehyde (MDA) content were determined according to previous studies with minor modifications (Li et al. 2021; Shan et al. 2022). Briefly, the CI index was visually scored by evaluating the degree to which the peel browning was characterized by grading of browning area proportion (Wang et al. 2014b). A Minolta chromameter CR-400 (Minolta Camera Co. Ltd.) was used to record color values at equidistant points around the middle part of the peel with three biological replicates, and the readings were expressed as L^*^ (lightness). For MDA measurement, 0.4 g frozen peel powder was homogenized with 1 mL of 10% (W/V) trichloroacetic acid (TCA). After centrifugation at 12, 000 × g and 24℃ for 20 min, the supernatant phase was collected. 0.4 mL of the supernatant were taken and then added to 1.2 mL of 0.5% thiobarbituric acid. The mixture was boiled for 20 min and the absorbances were determined at 450, 533 and 600 nm. The MDA concentration (µmol g^−1^) on a fresh weight (FW) basis was calculated according to the formula of {5 × [6.45 × (A532 – A600)] – 0.56 × A450} × V_t_ / (W × V_s_). V_t_, W, and V_s_ represent extract liquid volume, plant tissue fresh weight (g) and measured liquid volume, respectively. PPO, POD activity and H_2_O_2_ level were measured with reagent kit (Solarbio) according to operating instructions.

### RNA extraction, qrt-PCR and srna northern blot analysis

Total RNA extraction, qRT-PCR and sRNA northern blot was carried out as previously stated (Zhu et al. 2020). Reverse transcription for miRNA was performed using the miRNA 1st Strand cDNA Synthesis Kit (by tailing A) (Vazyme Biotech, Nanjing, China). For qRT-PCR, the banana *MaRPS2* was used an internal reference control and relative gene expression values were computed using the 2^−ΔΔCt^ method. U6 as the loading control for the northern blot analysis and RT-qPCR for miRNA. All primer sequences in this work are listed in Supplemental Data Set S1.

### Identification and phylogenetic analysis of maspl gene family

All amino acid sequences of bananas were downloaded from the banana genome database (Version 4.3). The SPL homologous amino acid sequence of *Arabidopsis* and rice to establish library reference sequence from *Arabidopsis thaliana* genome database (TAIR) (http://www.arabidopsis.org/) and UniPort database (http://www.uniprot.org/) were downloaded, respectively. The HMM file of the conserved domain of amino acid sequence SBP (PF03110) from the PFAM protein database (http://pfam.xfam.org/) was downloaded, and when the cutoff E-value is 0.01 and other parameters are set by default, banana genes were searched by hmmsearch of HMMER 3.2 software (http://www.hmmer.org/). Then, the homologous amino acid sequences of *Arabidopsis thaliana* and rice were taken as the reference sequence, and the cutoff E-value was set as e^−10^. The BLAST tool of TBtools software (Chen et al. 2020a) was used to further screen the banana genes. Finally, PFAM and NCBI CDD (http://www.ncbi.nlm.nih.gov/cdd/) databases were used to verify the conserved domain of all the amino acid sequences of target genes. ClustalX 2.0 was used for amino acid multi-sequence alignment, and the evolutionary tree was constructed by Mega-X, and the obtained evolutionary tree was post-processed and beautified by Evolview online software.

To determine which *MaSPL* genes are targeted by miR156, we used the cDNA sequence of *MaSPL*s to predict the miR156 target sites in TAPIR, a web server for plant microRNA target prediction (http://bioinformatics.psb.ugent.be/webtools/tapir) (Bonnet et al., 2010). The search parameters used in the TAPIR were as follows: score ≤ 5.0; free energy ratio ≤ 0.7. We visualized the interactions between the miR156 and the targeted *MaSPL*s genes using the Gene Structure Display Server (GSDS v. 2.0; http://gsds.cbi.pku.edu.cn/). Finally, Adobe Illustrator (AI) software was used to modify the image.

### miR156c-mediated cleavage of *MaSPL*s

To validate miR156c-mediated cleavage of *MaSPL4/5/25/42/44*, RNA Ligase-Mediated 5’-RACE (RLM-5’-RACE) analysis was conducted following the manufacturer’s instructions for the First Choice RLM-RACE Kit (AM1700, Invitrogen). 1 µg of mixed total RNA isolated from banana peel samples was used for ligating 5’RNA adaptors at 37 °C for 1 h before reverse transcription at 42 °C for 1 h. Gene-specific primers were designed to conduct nested PCRs (Supplemental Data Set S1), and PCR products were gel-purified, cloned into the pTOPO001 Blunt Simple vector (Genesand Biotech, Beijing, China) and sequenced.

In addition, transient co-expression assays in tobacco leaves were used to verify the targeting relationship. A 331-bp product containing the precursor sequence of miR156c (*pre-miR156c*) was amplified from banana genomic DNA and then cloned into pBI121 vector (between the *Sma* I and *Sac* I sites). The predicted target site of miR156c in *MaSPL4/5(42)/25/44* and the mutant target site were cloned into PMS4 vector (between *Xho* I and *Xba* I sites). Those two vectors were co-transformed into 7-week-old *N. benthamiana* leaves. The GFP fluorescent signals were observed three days after infiltration.

### Subcellular localization analysis

The *MaSPL4/5/25/42/44* encoding sequences without stop codon were cloned into pSAT6-EYFP vector. Fusion constructs and control vectors were transformed into *Arabidopsis* mesophylls protoplasts by pegylated transfection (Bonnet et al., 2010). After incubated at 22 °C under light for 18 h, Fusion constructs and control vectors were transformed into *Arabidopsis* mesophylls protoplasts by pegylated transfection (Leica SP8 STED 3X). The excitation wavelengths of YFP and mCherry are 514 nm and 590 nm, respectively. YFP and mCherry fluorescence were detected at 534 nm and 610 nm, respectively.

### Y1h assay

The miR528 promoter region containing three GTAC motifs (210 bp) was cloned into the pABAi vector to generate the reporter construct *miR528pro*::*AurR*. Firstly, pABAi-miR528pro plasmid was transferred to yeast and the Aureobasidin A (AbA) concentration inhibiting the growth of recombinant yeast was screened and determined as 200 mg/L. To generate the AD-MaSPL4/5/25/42/44, the full-length coding sequences of MaSPL4/5/25/42/44 were individually amplified using PCR and cloned into the pGADT7-AD vector, which contains Leu biosynthesis genes. The constructed and empty pGADT7-AD vectors were then transferred into yeast strains containing *miR528pro*::*AurR*, and the monoclones successfully transferred with AD vectors were cultured on YPDA solid medium containing AbA at the selected concentration.

### Dual-luciferase reporter system

The coding sequences of SPLs were subcloned into pGreenII 62-SK vector to produce the effector construct. The double reporter vector contains a GAL4-LUC in front of mini 35 S and an internal control REN driven by the 35 S promoter. To evaluate the binding activity of MaSPLs to the promoter of *MIR528*, MaSPLs were cloned into pGreenII 62-SK as effector plasmids (Hellens et al. 2005). The promoter of *MIR528* containing the GTAC motifs was inserted into pGreenII 0800-LUC as reporter plasmids (Hellens et al. 2005). Similarly, the promoter of *MaFAR4*, *MaKCS11*, *MaKCS20* and *MaPOD5* were also inserted into pGreenII 0800-LUC vector. The effector and reporter plasmids were co-transformed into tobacco leaves by *Agrobacterium* strain GV3101 (pSoup). Firefly LUC and REN activities were measured by the dual luciferase assay kit (Promega) after 3 d of co-transformation. The LUC/REN ratios were calculated to reflect the final transcriptional activity.

### Protein expression and purification, and emsas

The coding sequences of MaSPL4/5/25/42/44 were amplified using PCR, then inserted into the pGEX-4T-3 vector (Amersham Biosciences) and expressed as a glutathione S-transferase fusion protein (GST-SPLs) in Rosetta (DE3; ToloBio, Shanghai, China). The fusion proteins were purified using glutathione Sepharose 4B beads (GE Healthcare). Oligonucleotide probes containing GTAC motifs were synthesized with biotin (Sangon Biotech, Shanghai, China). An EMSA was performed using the Chemiluminescent EMSA Kit (Thermo Fisher Scientific).

### DAP-seq and RNA-seq analysis

Total RNA samples extracted from EV and OE-*MaSPL4* (14 days after cold treatment) banana fruits were used to construct libraries for RNA sequencing (RNA-seq). RNA-seq was carried out at Gene Denovo Biotechnology Co. (Guangzhou, China). DNA affinity purification sequencing (DAP-seq) was conducted as described in (Wei et al. 2022). The genomic DNA was extracted from banana leaf. The full-length cDNA of MaSPL4 was cloned into the expression vector and fused with a HaloTag.

## Supplementary Information

Additional file 1: Data Set S1. Primer sequences used in this study. Data Set S2. Cis-acting elements found in the promoter of banana *MIR528*. Data Set S3. List of characterized *MaSPL* genes in banana genome. Data Set S4. List of MaSPL4-binding peaks detected by DAP-seq. Data Set S5. List of putative MaSPL4-binding motifs identified by DAP-seq. Data Set S6. List of MaSPL4-binding genes identified from DAP-seq analysis. Data Set S7. List of DEGs identified by RNA-seq from OE-*MaSPL4* banana fruit. Data Set S8. List of MaSPL4-binding DEGs via integration of DAP-seq and RNA-seq data.

Additional file 2: Figure S1. Map of pRHV vector for transient transfer and fragment schematics of miR528 and miR156 silenced by STTM method. Figure S2. The 1.5 kb promoter sequence of banana *MIR528* with multiple cis-acting elements. Figure S3. Characterization and miR156 targeted analysis of *MaSPL*s. Figure S4. Expression pattern of *MaSPL*s in banana peel under cold stress. Figure S5. Overexpressing and silencing miR156c in banana transverse slices. Figure S6. MaSPL4 binding to the promoter regions of other *MIRNA* genes.

## Data Availability

Data supporting the findings of this study are available in the supplementary information.

## Abbreviations

Bp: Base pair
CBF: C-repeat binding transcription factor
CDS: Coding sequence
DAP-seq: DNA affinity purification sequencing
DEGs: Differentially expressed genes
DLR: Dual-luciferase reporter system
EMSA: Electrophoretic mobility shift
FAR: Fatty acyl-CoA reductase
FDR: False discovery rate
FW: Fresh weight
GFP: Green fluorescent protein
GO: Gene ontology
GST: Glutathione S-transferase
KCS: 3-ketoacyl-CoA synthase
KEGG: Kyoto encyclopedia of genes and genomes
LCR: Leaf curling responsiveness
LUC: Firefly luciferase
MDA: Malondialdehyde
miRNA: microRNA
NLS: Nuclear localization signal
NS: Not significant
OE: Overexpression
PPO: Polyphenol oxidase
POD: Peroxidase
qRT-PCR: Quantitative real-time polymerase chain reaction
RACE: Rapid amplification of cDNA ends
REDOX: Reduction-Oxidation
REN: Renilla luciferase
RNA-seq: RNA sequencing
ROS: Reactive oxygen species
RPS2: Ribosomal protein S2
SBP: SQUAMOSA promoter binding protein
SD: Standard deviation
SE: Standard error
SPL: SQUAMOSA-PROMOTER BINDING PROTEIN-LIKE
STTM: Short tandem target mimic
TCA: Trichloroacetic acid
UTR: Untranslated region
Y1H: Yeast one-hybrid
YFP: Yellow fluorescent protein
