# Supplementary material for: Transcriptional regulation of miR528-PPO module by miR156 targeted SPLs orchestrates chilling response in banana
Source: Mol Hortic. 2025 Jan 10;5:2. doi: 10.1186/s43897-024-00115-1 (PMC11720309; doi:10.1186/s43897-024-00115-1)
Supplement: Supplementary file 2 — Additional file 2: Figure S1. Map of pRHV vector for transient transfer and fragment schematics of miR528 and miR156 silenced by STTM method. Figure S2. The 1.5 kb promoter sequence of banana MIR528 with multiple cis-acting elements. Figure S3. Characterization and miR156 targeted analysis of MaSPLs. Figure S4. Expression pattern of MaSPLs in banana peel under cold stress. Figure S5. Overexpressing and silencing miR156c in banana transverse slices. Figure S6. MaSPL4 binding to the promoter regions of other MIRNA genes. [file 43897_2024_115_MOESM2_ESM.docx]

­­­­
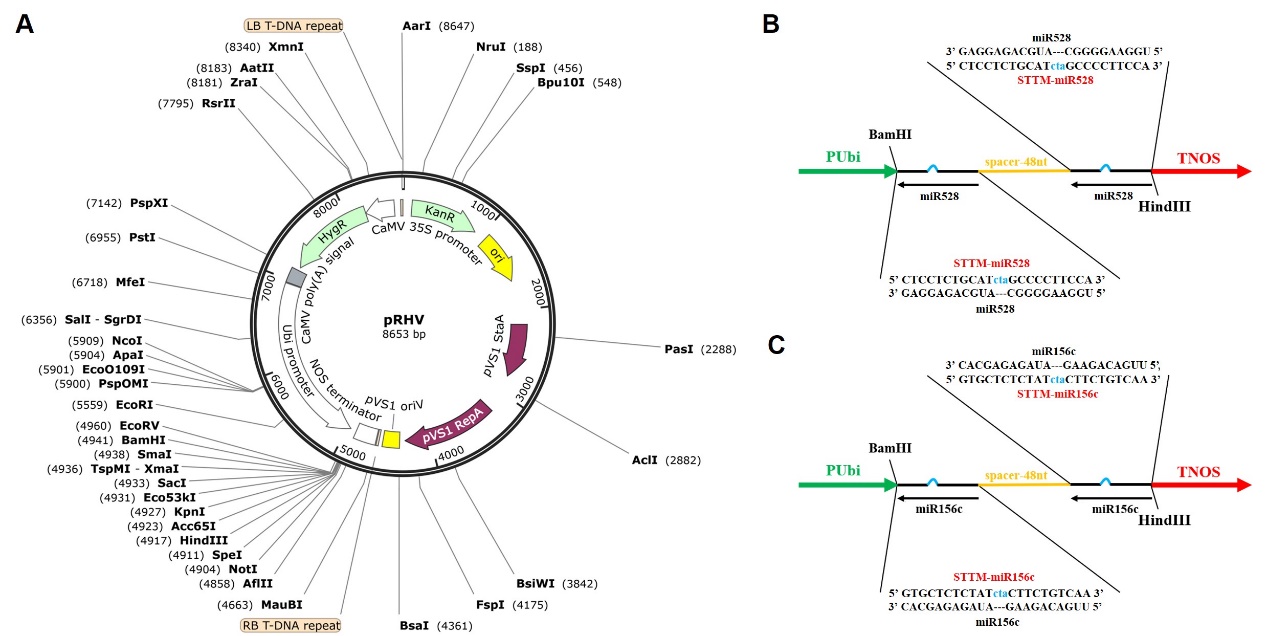


**Figure S1** Map of pRHV vector for transient transfer (A) and Fragment schematics of miR528 (B) and miR156c (C) silenced by STTM method.

**Figure S2** The 1.5kb promoter sequence of banana *MIR528* with multiple cis-acting elements. The cis-regulatory elements are in the box with their names indicated above the box.


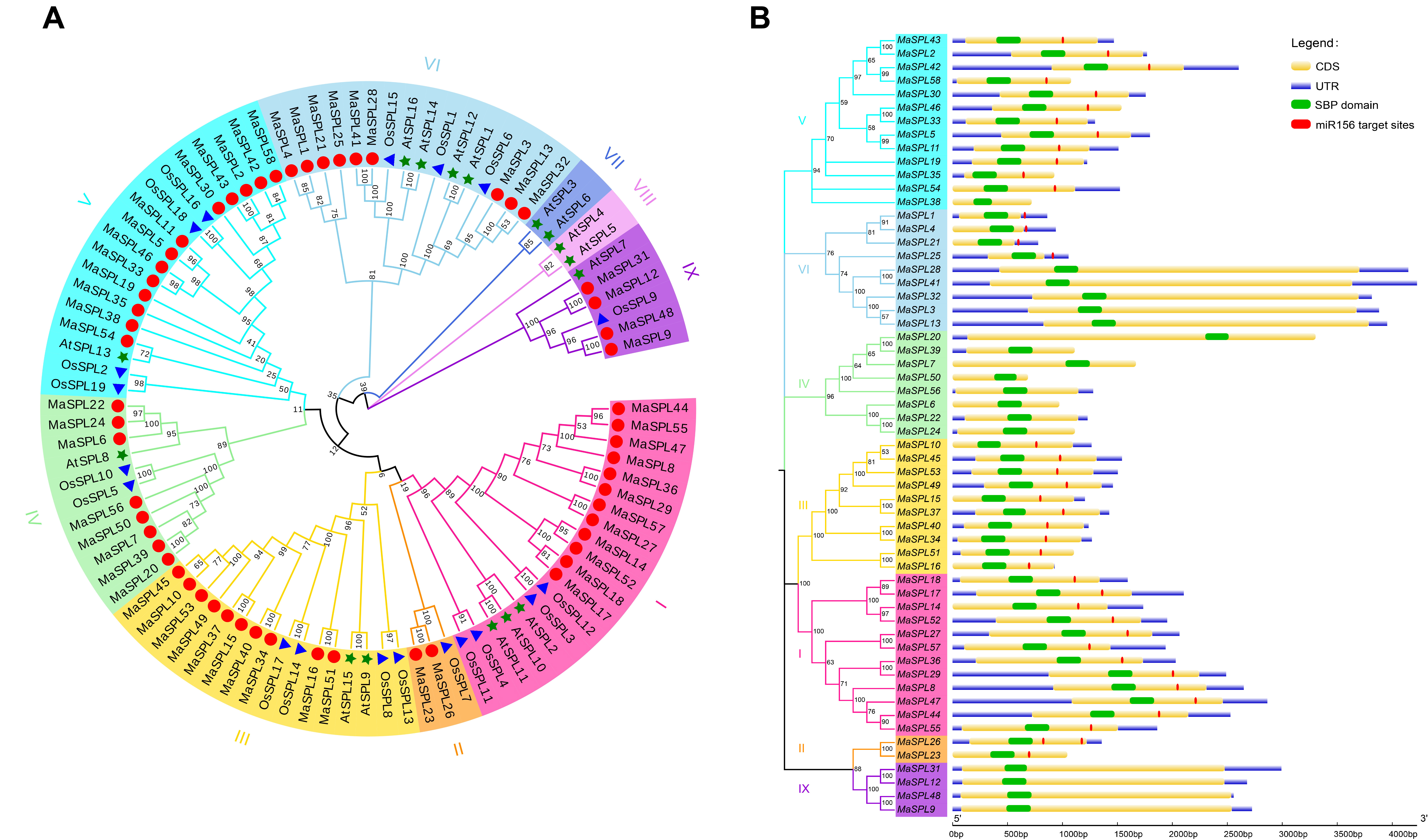


**Figure S3** Characterization and miR156 targeted analysis of *MaSPL*s. (A) Phylogenetic tree and classification of *MaSPL/OsSPL/AtSPL* genes. The tree was constructed based on the SPL protein sequences of *Arabidopsis thaliana*, *Oryza sativa*, and banana. The red circles, green stars and blue triangles represent members of the SPL family of banana, *Arabidopsis thaliana* and *Oryza sativa*, respectively. (B) Prediction of miR156 target sites of *MaSPL*s. The yellow, blue, green and red structures represent the coding sequence (CDS), the 5' and 3' untranslated region (UTR), the SBP conserved domain and the target site of miR156, respectively.


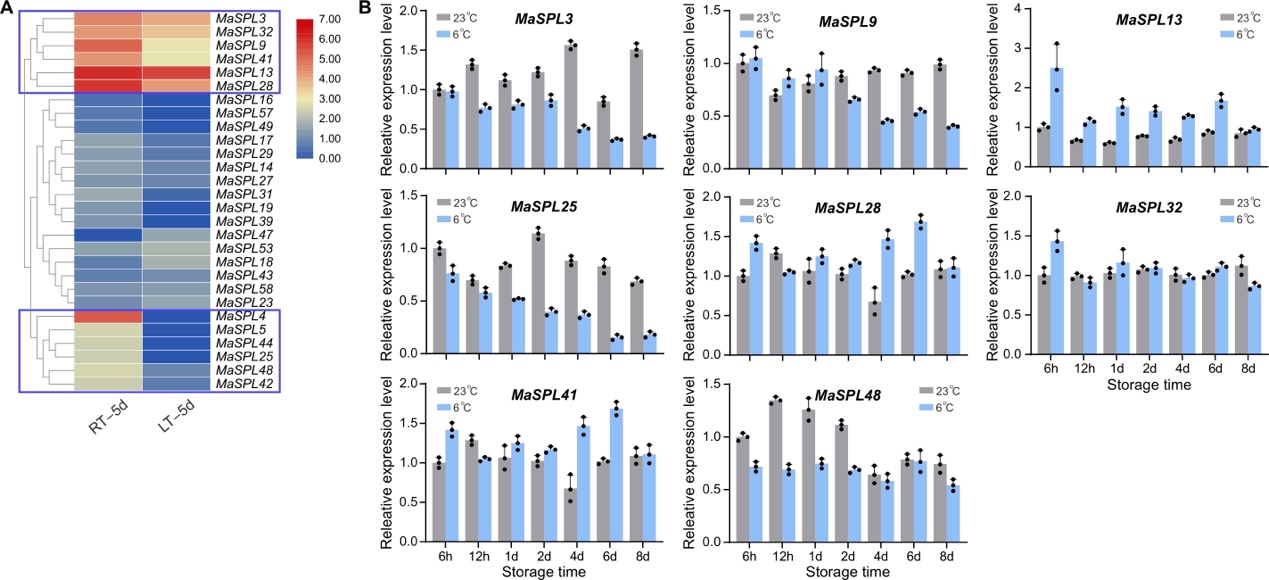


**Figure S4** Expression pattern of *MaSPL*s in banana peel under cold stress. (A) Expression profiling of expressed *MaSPL* gene family members in peel of banana, based on previously published RNA-seq datasets. RT means 25℃ at room temperature and LT means 6℃ under low temperature stress. Highly expressed *MaSPL*s are in the blue box. (B) Gene expression pattern of some highly expressed *MaSPL*s in banana peel during cold stress. The *RPS2* gene was used as an internal reference. Data are presented as the means ± SD of three biological replicates (n = 3).


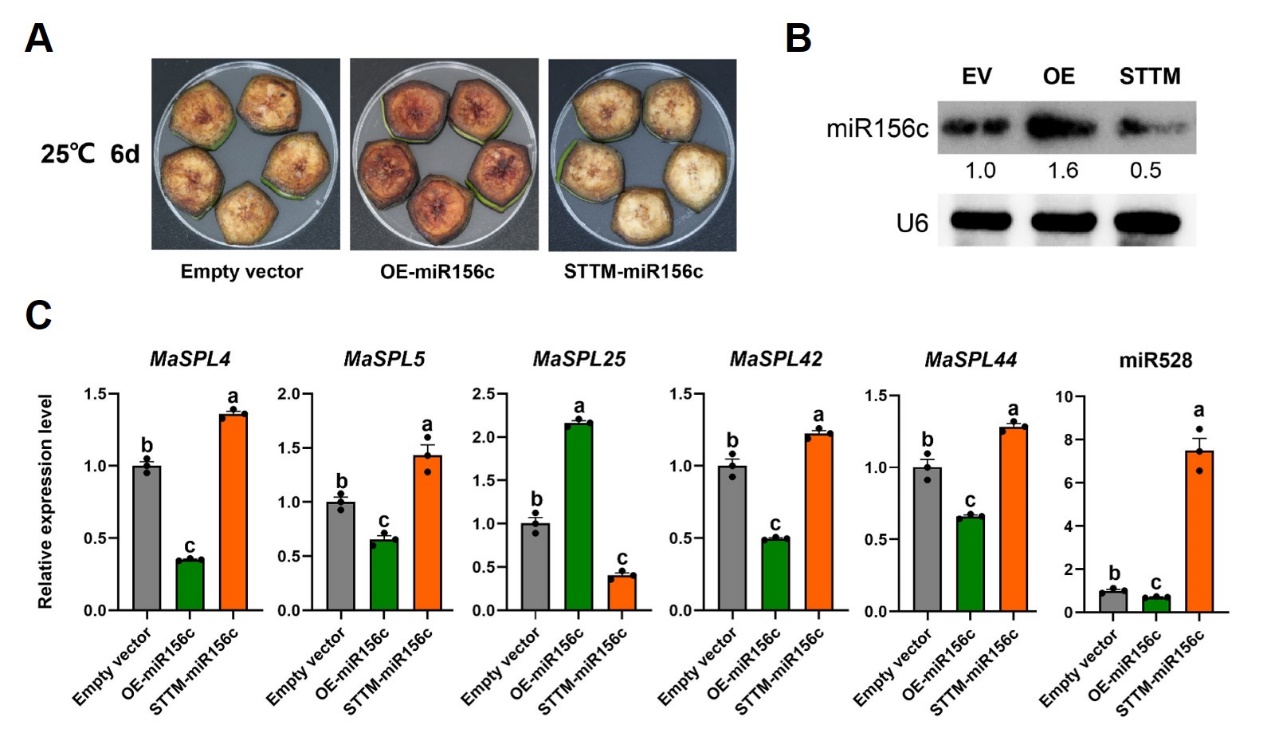


**Figure S5** Overexpressing and silencing miR156c in banana transverse slices. (A) Appearance of overexpressing and silencing miR156c in banana slices. (B) Abundance of miR156c tested by northern blot. U6 was probed as a loading control. (C) Expression pattern of *MaSPL4/5/25/42/44* and miR528. The *RPS2* gene and U6 were used as an internal reference, respectively. Data were presented as the means ± SD (n = 3) and significant differences were indicated with different letters at 0.05 level, as determined by one-way ANOVA.


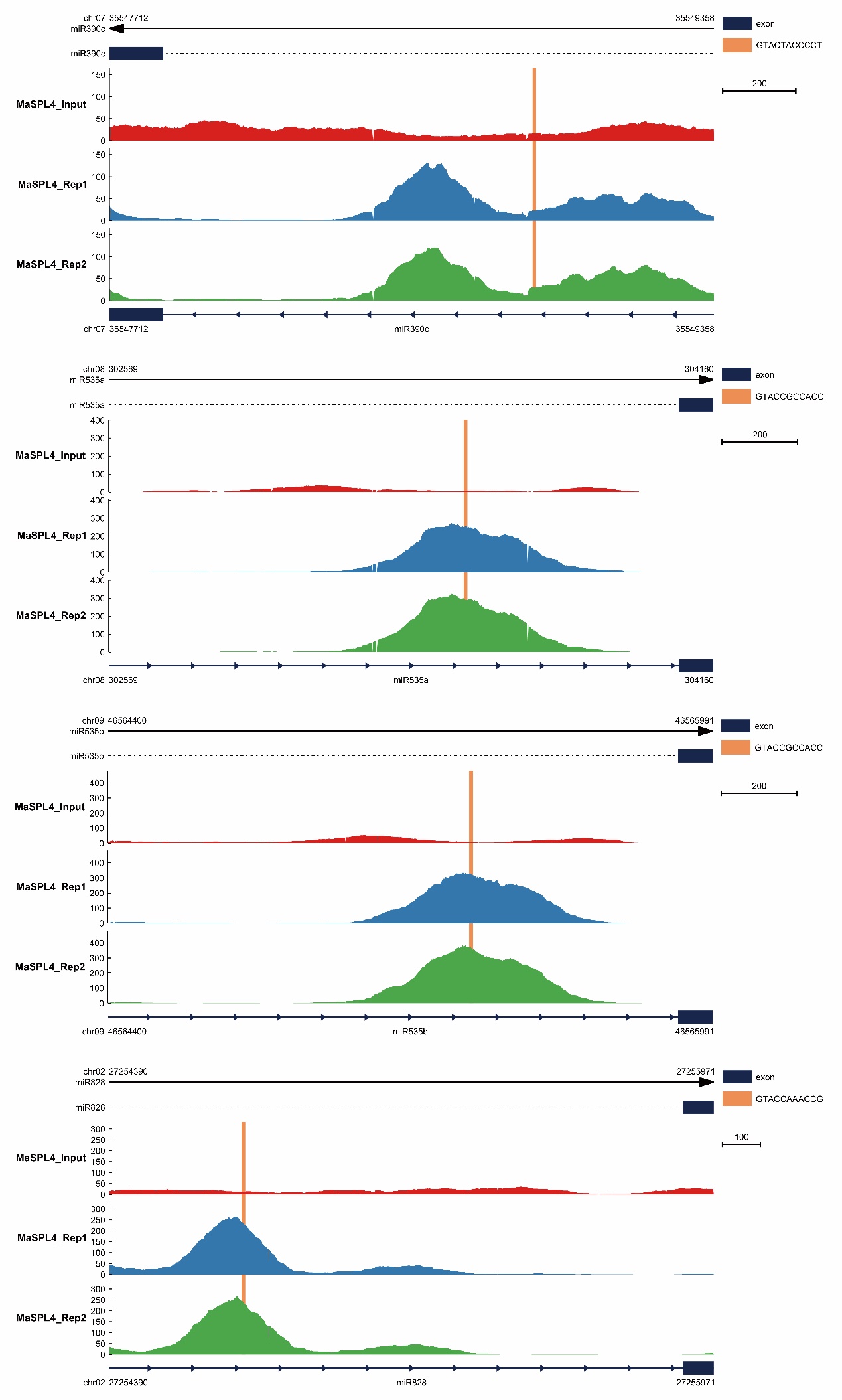


**Figure S6** MaSPL4 binding to the promoter regions of other *MIRNA* genes. Binding peaks of MaSPL4 in the promoter of *MIR390c*, *MIR535a*, *MIR535b* and *MIR828* identified by DAP-seq. Both miRNA precursor and upstream 1.5 kb regions are shown. The arrows indicate the direction of *MIRNA* gene transcription and the solid orange lines indicate the positions of MaSPL4 binding motif.
